# Supplementary material for: The kinematics of cyclic human movement
Source: PLoS One. 2020 Mar 5;15(3):e0225157. doi: 10.1371/journal.pone.0225157 (PMC7058299; doi:10.1371/journal.pone.0225157)
Supplement: Attachment — Submitted filename: PLOS review.docx [file pone.0225157.s001.docx]

In this work, the authors developed a mathematical model to describe cyclic human movement. They then collected data from both running and biking, and fit the model based on subject-specific measurements. Simulation of the fitted models generates movements that are quite similar to measurement, suggesting that the model is able to, at least generally, describe the motion. Overall, I have no major reservations about the validity of this work but have a number of recommendations that would improve the overall readability of this paper.

Major Comments

- The introduction and discussion are particularly hard to follow. I highly recommend that the authors reorganize and separate these sections into individual paragraphs.
- The method section would benefit from a brief overview paragraph, describing the steps (subheadings) and how they fit together.
- In the ‘model’ subsection, the authors should pay careful attention and ensure that all symbolic terms are clearly defined when the first appear. In particular, I noted that K (eq1), a (eq2) and a_0_ and a_1_ were not defined when they first appear.
- The results section, as it is right now, is quite anemic. Rather than simply listing figures with their minimal captions, It would be beneficial to write full, organized paragraphs describing these results with reference to figures in-text.
- On a similar note, many of the figures would benefit from a legend rather than relying on the caption alone to describe what the colors mean.

Minor comments

- There are a few cases where the wrong word is used. On page 5, I believe the authors meant to say ‘cycle’ instead of ‘circle’, on page 4 I think they meant ‘insight’ instead of ‘inside’.
- I would appreciate if the authors could more fully discuss where this type of research is going. What is the utility? I assume the intent is to compare individuals to try to identify characteristics indicative of good performance or pathology?
