# Supplementary material for: The kinematics of cyclic human movement
Source: PLoS One. 2020 Mar 5;15(3):e0225157. doi: 10.1371/journal.pone.0225157 (PMC7058299; doi:10.1371/journal.pone.0225157)
Supplement: Attachment — Submitted filename: Response to Reviewers.docx [file pone.0225157.s002.docx]

Following, please find our responses to your remarks directly after each of your statements marked in red.

When submitting your revision, we need you to address these additional requirements.

1. Please ensure that your manuscript meets PLOS ONE's style requirements, including those for file naming. The PLOS ONE style templates can be found at
   http://www.journals.plos.org/plosone/s/file?id=wjVg/PLOSOne_formatting_sample_main_body.pdf and <http://www.journals.plos.org/plosone/s/file?id=ba62/PLOSOne_formatting_sample_title_authors_affiliations.pdf>

We checked these requirements thoroughly and adjusted the style where needed. Further we checked all figures with the provided graph tool.

2. Your ethics statement must appear in the Methods section of your manuscript. If your ethics statement is written in any section besides the Methods, please move it to the Methods section and delete it from any other section. Please also ensure that your ethics statement is included in your manuscript, as the ethics section of your online submission will not be published alongside your manuscript.

 The ethics statement was moved to the end of the Methods section.

4. Is the manuscript presented in an intelligible fashion and written in standard English?

PLOS ONE does not copyedit accepted manuscripts, so the language in submitted articles must be clear, correct, and unambiguous. Any typographical or grammatical errors should be corrected at revision, so please note any specific errors here.

Reviewer #1: No

Reviewer #2: Yes

Reviewer #3: Yes

The text was reviewed with our cooperation partners at the Northern Michigan University (USA) to address the typographical and grammatical errors. Further we restructured our introduction and discussion part for a better readability. We added a conclusion part at the end of the text. There we concluded the model’s idea, highlighted practical applications and future directions.

**Reviewer 1)** The authors have provided intensive mathematical models without a proper prove and rational for their selection. In addition, the equations are poorly represented with many undefined elements across the paper. This highly affects the readability of this manuscript and is necessary to be revised thoroughly.

Now all elements are defined at their first appearance within the text. We elaborated on the rationality for the equation. While the attractor concept was of our 2013 paper, an important new feature were the random processes with its normal or unit distributions.

2) Abstract and Results: no overall quantified accuracy/performance result (e.g. averaged across all subjects) is reported to compare the simulations and measurements.

The results (and also the Abstract) section was reviewed extensively. We extended the written parts, reworked the figures and added also general outcomes, like an overview of the defined nine constants and concluding numbers for the transient effect and morphing. These adjustments allow a better generalization of our approach and give an idea of the magnitude of the variables.

3) Simplification of Equation 14 is vague and no mathematical proof is provided.

The explanation for the simplification was rephrased. Noise is canceled out because of its normal distributed character with a mean of zero. So, the contributions cancel. The fluctuation has the characteristic of a “random walk”. Therefore, it can in principle make a big contribution. The correction term, however, brings the numbers back close to zero. Multiple of these sequences reduce the average contribution to give a small, neglectable contribution only.

4) The simulation app (TrackSimulator) seems to have problem in running at least on my computer (macOs).

We ran the app on different hardware, either on windows or macOS. On rare occasions we experienced problems. A mac with an intel processor should work. But, the necessary runtime libraries (about half a GB) must be downloaded with the installation. Here it is not enough to use runtime libraries, which might be on the system, since with each MATLAB update libraries change.

5) Super Attractor section: what does (minute x attractor time/acceleration/deviation/standard error/gyroscope data x average number of attractor points) show? Is it a vector? It is confusing.

The Super Attractor section was reviewed completely. We guess that the definition and idea of the super attractor comes across now.

6) Eq. 2: the authors have claimed that they used a fixed number of data points instead of a fixed time interval. This shows that the sampling frequency was not consistent otherwise this should not matter. Please explain.

The attractor is calculated from a one-minute data sample. These are about 60 to 80 cycles. So, there are cycles of different durations, having different numbers of data points. We calculate the average number n of data points within the cycles and adjust all cycles to have n data points. By averaging these cycles, we get an attractor with the average number of data points, hence having the original sampling frequency.

7) Eq. 12: how do you define the direction of these vector systems? The functions are in terms of “t” which is a scalar and no geometrical direction is introduced. This is a bit confusing.

First $T_{\parallel}$ is a function of time t and it is the deviation from the morphed attractor. We calculate the parallel component only using the Levenberg-Marquardt algorithm for the results of equation (14). In the simulation this is one of the input parameters.

8) Low quality figures. Fig. 2: what quantity does it show? Axis names are not visible.

The figure was redone and checked for visibility of the axe’s names. It gives a first qualitative comparison between measurement and simulation.

9) Fig. 6: how is the super attractor function identified? From the same data that are shown in the figure? How do you reproduce the function and prove its functionality in different walking conditions, e.g. walking up/down the stairs?

We rephrased the definition of the super attractor.

This study is concerned with running on flat ground only.

10) Table 3: why are there many empty cells in this table? No transient effect present? Please explain.

We filled the empty cells with No TE, for “No Transient effect” when there was no transient effect for the referred cell. This was the case for subjects, who showed no exponential behavior at the beginning of their running or cycling session.

11) The organization of paper is poorly handled as there are only one paragraph for introduction and discussion! Furthermore, I found several grammatical issues throughout the manuscript including:

The text was reviewed with our cooperation partners at the Northern Michigan University (USA) to address the typographical and grammatical errors. Further, the introduction as well as the discussion part was divided into appropriate sub sections for a better readability.

a. Model-number 1-line 9: circle -> cycle? Was corrected by the authors
b. Model-number 6-line 2: real live -> real life? Was corrected by the authors
c. Introduction-line 35: gives inside -> gives insight? Was corrected by the authors

12) The participants were regarded as athletes in the paper, but the general requirement of recruiting subjects does not reflect them being an athlete.

We labelled them now as “**recreational** athletes” to highlight their sportive status.

**Reviewer #2:** The purpose of the paper is to expound a model of cyclic human behavior. It is evaluated for 10 individuals, 5 for running and 5 for cycling. The major part of the work is the evaluation of the simulated model.

The readability of the results leaves a lot to be desired as from some figures (3,4) it is impossible to see a lot.

The results section was reviewed extensively. We extended the written parts, reworked the figures and added also general outcomes, like an overview of the defined nine constants and concluding numbers for the transient effect and morphing.

A clear conclusion is missing. While there is a discussion present, a clear conclusion is not given and therefore the reader is left alone to ponder a lot of questions set up in the discussion, and to come to the conclusion by oneself. This is what is most missing – a conclusion saying that the model is better than some other models and that it allows for something that was previously not allowed – all stated explicitly. Also, this implies some comparison, which is not present.

We addressed these remarks extensively in a new Conclusion section at the end of the text. There we concluded the model’s idea, highlighted practical applications and future directions.

Dynamical systems are not at all discussed in the paper, or just barely. Also, working with the phase of the motion would help a lot. Also, some deep neural networks approach that can be used to classify people based on data, and therefore somehow probably also predict the data are not mentioned, but surely they are out there.

We are aware that our model constitutes a dynamical system. Since dynamical systems are well known, we did not want to elaborate on it or give a definition. Also, we do cite a paper Janssen et al. [18] using neural nets, hence deep machine learning. Here we added newly the expression beside the term neural nets.

Given the amount of work (measurements) put into this paper, it is a bit too bad that it lacks in the presentation and comparison, but it is what still gives a chance to the paper.

The discussion is extended, now including the “characterizing constants”, which being used in the simulation. This allow the comparison between measurement and simulation.

Level of English could be improved (is not bad, but at some sentences I was really scratching my head wondering what was meant)

The text was reviewed with our cooperation partners at the Northern Michigan University (USA) to address the typographical and grammatical errors.

Some specific comments
Abstract starts with some claims on “roughly be divided into the categories theory or data driven”, which are totally unfounded – ok, citing is bad in the abstract, but then write it differently. Such claims – without data or at least citations, are totally useless and just say: I said this, now believe it! Not good practice

This part is reformulated.

and how such a developed model can be tested? -> I really do not understand this sentence and what you are trying to ask yourself

This part was rewritten for a better understandability. It can be found in the third paragraph of the introduction:

(…)Modern quantitative scientific endeavors to understand the mechanism behind the central movement trait already began as early as in the nineteenth century [16]. Describing cyclic motion most often is realized by selected specific body markers and their coordinate portrayal as function of time [17]. (…)

No wonder that -> this does not seem as an appropriate form for a scientific paper…

This was rewritten by the authors (see above)

this model does allow -> ALLOWS

Was corrected by the authors

There exist two types of models –> two types of models of what?

This was rewritten by the authors: There exist two types of **models describing human cyclic motion** - theory driven and data driven [18] – both with its own strong and weak aspects.

What are the colors of the arrows in Fig 3 and 4? And they don’t really match for some subjects…

We added a legend at the bottom of Figs 3 and 4 to better understand the components of the graphs. Furthermore, we adjusted the captions.

**Reviewer #3:** In this work, the authors developed a mathematical model to describe cyclic human movement. They then collected data from both running and biking, and fit the model based on subject-specific measurements. Simulation of the fitted models generates movements that are quite similar to measurement, suggesting that the model is able to, at least generally, describe the motion. Overall, I have no major reservations about the validity of this work but have a number of recommendations that would improve the overall readability of this paper. Please see my attachment.

Major Comments

- The introduction and discussion are particularly hard to follow. I highly recommend that the authors reorganize and separate these sections into individual paragraphs.

The introduction as well as the discussion part was divided into appropriate sub sections.

- The method section would benefit from a brief overview paragraph, describing the steps (subheadings) and how they fit together.

We wrote a whole new overview paragraph for our Methods section to get an idea of its components/subheadings and how all parts relate to each other.

- In the ‘model’ subsection, the authors should pay careful attention and ensure that all symbolic terms are clearly defined when the first appear. In particular, I noted that K (eq1), a (eq2) and a_0_ and a_1_ were not defined when they first appear.

Missing definitions were included.

- The results section, as it is right now, is quite anemic. Rather than simply listing figures with their minimal captions, It would be beneficial to write full, organized paragraphs describing these results with reference to figures in-text.

The results section was reviewed extensively. We extended the written parts, reworked the figures and added also general outcomes, like an overview of the defined nine constants and concluding numbers for the transient effect and morphing.

- On a similar note, many of the figures would benefit from a legend rather than relying on the caption alone to describe what the colors mean.

We added a legend at the bottom of the figure where appropriate to better understand the components of the graphs. Furthermore, we adjusted the captions.

Minor comments

- There are a few cases where the wrong word is used. On page 5, I believe the authors meant to say ‘cycle’ instead of ‘circle’, on page 4 I think they meant ‘insight’ instead of ‘inside’.

All of these were corrected by the authors. Further the text was generally reviewed with our cooperation partners at the Northern Michigan University (USA) to address the typographical and grammatical errors.

- I would appreciate if the authors could more fully discuss where this type of research is going. What is the utility? I assume the intent is to compare individuals to try to identify characteristics indicative of good performance or pathology?

We addressed these remarks extensively in a new Conclusion and discussion section at the end of the text. There we concluded the model’s idea, highlighted practical applications and future directions.
